# Y-STR Haplogroup Diversity in the Jat Population Reveals Several Different Ancient Origins

**DOI:** 10.3389/fgene.2017.00121

**Published:** 2017-09-20

**Authors:** David G. Mahal, Ianis G. Matsoukas

**Affiliations:** ^1^School of Sport and Biomedical Sciences, University of Bolton Bolton, United Kingdom; ^2^Extension Division, University of California, Los Angeles Los Angeles, CA, United States

**Keywords:** Y-chromosome, Y-DNA, Y-STR, haplotypes, haplogroups, India, Jats, Pakistan

## Abstract

The Jats represent a large ethnic community that has inhabited the northwest region of India and Pakistan for several thousand years. It is estimated the community has a population of over 123 million people. Many historians and academics have asserted that the Jats are descendants of Aryans, Scythians, or other ancient people that arrived and lived in northern India at one time. Essentially, the specific origin of these people has remained a matter of contention for a long time. This study demonstrated that the origins of Jats can be clarified by identifying their Y-chromosome haplogroups and tracing their genetic markers on the Y-DNA haplogroup tree. A sample of 302 Y-chromosome haplotypes of Jats in India and Pakistan was analyzed. The results showed that the sample population had several different lines of ancestry and emerged from at least nine different geographical regions of the world. It also became evident that the Jats did not have a unique set of genes, but shared an underlying genetic unity with several other ethnic communities in the Indian subcontinent. A startling new assessment of the genetic ancient origins of these people was revealed with DNA science.

## Introduction

### Population and demographics

The Jats represent one of the largest ethnic groups that has evolved in the northwest region of the Indian subcontinent—India and Pakistan—over several thousand years. Since the partition of India in 1947, Hindu and Sikh Jats have lived primarily in India, and the Muslim Jats have lived primarily in Pakistan.

In 2012, the Jat population in India—mostly Hindus and Sikhs—was reported to be 82.5 million people (Chatterji, 2012). The last time the population was surveyed according to caste–in the 1931 Census of India–the Jats belonged to three main religions: Hinduism 47%, Islam 33%, and Sikhism 20% (Burdak, 2016). Assuming the ratio among religions has stayed about the same (i.e., 33% for Islam and 67% combined for Hinduism and Sikhism), the population of Muslim Jats in 2012 can be extrapolated to about 40.6 million (82.5 million/67 × 33). On this basis, the total population of all Jats in the Indian subcontinent is estimated to be around 123 million people, roughly equal to the combined population of France, Spain, and Portugal.

### Archeological evidence

The origins of the Indus Valley Civilization—also known as the Harrapan Civilization—can be traced to 7,380–6,201 BCE in northwestern India (Khandekar, 2012). A recent discovery of a large Indus Valley site was made in Rakhighari, about 160 km from New Delhi. Its origins go back to about 5000 BCE (Subramanian and Khan, 2016). This ancient civilization flourished in the third millennium BCE (Harari, 2015), and its people were known as the earliest agriculturists in South Asia (Harris, 1996).

Originally the Jats were pastoralists (Khazanov and Wink, 2001), and gradually became farmers. Although farming settlements emerged in the Indus Valley Civilization about 4,000 BCE (Violatti, 2013), and Jats have been firmly settled as agriculturists in the same geographical region, a connection between the two has not been explored thoroughly. Apparently, this is because there is no conclusive written history of the people of the Indian subcontinent when we look back more than about 2,500 years. As a result, the deep ancestry of the Jat people has remained a mystery for a long time.

### Historical perspectives

Among the earliest available books from India—written in Sanskrit—that provide some glimpses of history are the Rigveda, composed between 1,500 and 500 BCE (Flood, 1996), and the Mahabharata, composed between 400 BCE and 400 CE (Molloy, 2008). This textual evidence contains some references to the existence of agriculture in the area, and mentions people known as the *Srinjaya*—meaning, sons of the sickle or farmers (Hewitt, 1894).

Some early Greek and Roman historians had acquired fragments of information about India from soldiers and merchants in the Persian Empire. But there is no reliable written history of the Indian subcontinent before Alexander the Great's campaign of India in 327 BCE (Smith, 1921). Although archeology has shed some light about the distant past—and even this record is incomplete—written history of India goes back only about 2,500 years.

More recently, numerous books have been written about Indian history and scholarship has been attempted over the origins of the Jats. Several historians have asserted that Jats were descendants of Indo-Aryans (Risley, 1915; Vaidya, 1921; Singh, 1963; Joon, 1967; Dahiya, 1980; Jindal, 1992; Qanungo, 2003), or Indo-Scythians (Elphinstone, 1841; Cunningham, 1871; Tod, 1920; Mahil, 1955; Marshall, 1960; Dhillon, 1994; Nijjar, 2008). The focus of most historians has been on the Indo-Aryan migrations to north India, which started around 1750 BCE, and the arrival of Indo-Scythians later around 200 BCE. The historical debate between the Aryan and Scythian origins of the Jats has continued (Panwar, 1993). In the scientific community as well, there are varied opinions regarding the Indo-Aryan migrations to India (Wells et al., 2001; Cordaux et al., 2004; Metspalu et al., 2011).

### A pioneer study based on ethnography

In the early days of anthropology, craniometry seemed to offer a solution to the study of antiquity of humans, and attention was directed mainly at the examination of skulls that were excavated. This led to anthropometry, a process of measuring various parts of living humans. Sir Herbert Risley, who was in-charge of the Census of India, introduced anthropometry in India in 1886, and became a pioneer in the application of scientific methods to classify ethnic groups of the country. Based on their tall stature, a long head, fair complexion, and narrow nose, the Jats were classified as Indo-Aryan, and groups with a medium stature, a broad head, fair complexion, and a moderately fine nose, were classified as Scytho-Dravidian (Risley, 1915). The study received criticism, but it opened new fields of enquiry about the people of the subcontinent.

### Tracing deep ancestry

We can identify our progenitors going back a few hundred years with traditional genealogical methods using records of family history. Beyond that, tracing ancestry is complicated because there is generally no documentation. New methods are now available based on recent developments in DNA science. Because DNA is inherited from our parents, it is possible to track the genes going back thousands of years and determine where our ancestors came from. Genetic tests allow us to trace the origins and paths of ancestors.

In DNA testing, two kinds of markers on the DNA strand are assessed: short tandem repeats (STRs), and single nucleotide polymorphisms (SNPs). The STRs are found on the Y-chromosome (Y-STRs) and used exclusively for tracing male lines of heredity. The SNPs are found on the Y-chromosome and in MT-DNA. They are used to trace male and female lines of heredity. The result of the test is a set of numbers, referred to as the *haplotype*, which is used to identify the haplogroup of an individual. Thus, the *haplogroup* represents a group of people who have inherited common genetic characteristics from the same most recent common ancestor (MRCA). All humans belong to haplogroups which are designated according to their Y-DNA and MT-DNA. The geographic origins of a Y-chromosome haplogroup can be deciphered from the phylogenetic tree of mankind maintained by the International Society of Genetic Genealogy (ISOGG, 2016).

By identifying Y-chromosome haplogroups and their geographic origins, this study has shown that: (a) the genetic origins of the Jats can be traced to at least nine different ancestors and geographical areas of this world, and (b) as a result, this ethnic group did not emerge from a single ancient population such as, the Indo-Aryans or Indo-Scythians.

## Materials and methods

### Haplogroups

The nonrecombining portion of the human Y-chromosome is paternally inherited, and passes from father to son essentially unchanged. But occasionally a random change known as a polymorphism or mutation occurs. Such mutations—also called markers—serve as beacons and can be mapped. When geneticists identify a mutation in a DNA test, they try to determine when it first occurred and in which geographic region of the world. Thus, the Y-chromosome haplogroup can be used to trace the paternal line of the individual (Jobling and Tyler-Smith, 2003). The Y-DNA tests are available only for men.

Because Y-DNA haplogroups are closely linked to geography and populations, they serve as important genetic indicators to trace paternal lineages and their ancient origins. This study has relied on the Y-DNA *haplogroup* as the primary gauge for exploring deep ancestry of the MRCAs of the Jats.

### Y-DNA haplogroup tree

The Y-DNA haplogroups contain many branches called subhaplogroups or subclades and form a phylogenetic tree of mankind. The branch lengths of the tree are governed by the mutation rates of Y-STRs and Y-SNPs. The markers on the phylogenetic tree provide pieces of evidence regarding the date and geographical origin of the MRCA in the distant past. The top-level haplogroups are identified by letters, A through T. Their subhaplogroups or subclades are expressed as letters and numbers (G2, R1b1, E3b1b, etc.). The tree is updated periodically according to new developments in the field. This study has relied on the Y-DNA *haplogroup tree* (ISOGG, 2016) to identify the geographical origins of the Jats.

### Identifying haplogroups

Two different methods are incorporated in this study to determine haplogroups. One method examined SNPs on the Y-chromosome in the laboratory with actual DNA samples of men. Another method examined STRs in the Y-chromosome haplotypes found in published literature. For these records with Y-STR profiles, a software program was used to predict the haplogroups.

Several software tools are available that use Y-chromosome haplotypes to identify haplogroups. These software tools are based on mathematical calculations. A study of a software tool, Haplogroup Classifier, developed at the University of Arizona showed that by using machine learning algorithms and data derived from a set of Y-linked STRs, it was possible to assign Y-chromosome haplogroups to individual samples with a high degree of accuracy (Schlecht et al., 2008). The software tool yHaplo was developed at 23andMe (23andme, 2017), a DNA testing company, to enable researchers to identify the Y chromosome haplogroups of males in a genetic sample. The software has been run on more than 600,000 males in the 23andMe database to confirm haplogroup calls for several hundred individuals (Poznik, 2016). In this study, Whit Athey's Haplogroup Predictor software was used (Athey, 2006).

### Datasets

Two separate datasets were created for this study, one representing the Jat population, and one representing 38 other ethnic groups of the Indian subcontinent for comparison purposes.

For the Jat population, a dataset of 302 men was compiled consisting of 44 records from the Genographic Project database (Genographic, 2016), with permission of the National Geographic Society, and 258 records from published sources (Henke et al., 2001; Nagy et al., 2007). The haplogroups in the Genographic Project database were already predetermined at source, based on examination of SNPs in the lab with actual Y-DNA samples. The records from published sources contained haplotypes with nine to twelve Y-STR loci (DYS19, DYS385a, DYS385b, DYS389-1, DYS389-2, DYS390, DYS391, DYS392, DYS393, DYS437, DYS438, and DYS439) on the Y-chromosome. The haplogroups for these 258 records were identified by processing their haplotypes in the Haplogroup Predictor software. Only the predominant top-level haplogroups were identified (the subclades or subhaplogroups were not used). All haplogroups from the Genographic Project database and published sources were merged and sorted in Excel. This dataset of 302 records represented 294 Jats from India and eight from Pakistan. The Muslim Jats were under represented in the sample.

For other ethnic groups of the Indian subcontinent, a dataset of 1,855 men representing 38 ethnic groups in Bangladesh, India, and Pakistan was compiled from the Genographic Project database (Genographic, 2016), and published sources (Sengupta et al., 2006; Zhao et al., 2009; Giroti and Talwar, 2010; Nair et al., 2011; Chennakrishnaiah et al., 2013; Lee et al., 2014). All haplogroups for these records were predetermined at source, based on examination of SNPs in the lab with actual Y-DNA samples. For this dataset as well, all haplogroups from the Genographic Project database and published sources were merged and sorted in Excel.

### Comparison with foreign populations

Migrations and invasions have been recurring themes in the history of the Indian subcontinent. To corroborate a nexus with other populations, the genetic relationship of the Jat population was compared with 25 populations of Central Asia and Northern Europe, comprising 21,899 Y-STR haplotypes in the world population database maintained at YHRD.org (see sources of data under Supplementary Information). A sample of 258 Y-STR haplotypes of Jats was used. The calculations were performed with AMOVA and MDS online software tools provided at the YHRD website http://www.yhrd.org.

AMOVA (analysis of molecular variance), is a statistical program for estimating population differentiation directly from molecular distance among DNA haplotypes (Excoffier et al., 1992). This online tool at YHRD.org provides an option to use the R_st_ or F_st_ measure—they are analogous—to determine the proportion of variance between populations. The R_st_ measure was used because it is reported to provide relatively unbiased estimates of population divergence, whereas the F_st_ measure tends to show too much population similarity (Slatkin, 1995). A multidimensional scaling (MDS) plot based on a non-metric algorithm (Kruskal, 1964) was also created, to provide a visual representation of the pattern of similarities or distances between Jats and 11 foreign populations. The full set of 25 populations from the AMOVA calculation was not used in the MDS plot to avoid overlapping of labels.

## Results

### Genetic distance

The results of the AMOVA and MDS tests (Table 1) confirmed that the Jats had genetic affinities with several foreign populations and provided some insights into their genetic makeup. The genetic difference between the Jats and the tested populations ranged from a small distance of 0.0257 in Afghanistan to a larger distance of 0.2128 in FYR Macedonia.

**Table 1:** Analysis of molecular variance (AMOVA): Pairwise Rst genetic distance between Jats and 25 selected population and a multidimensional scaling (MDS) plot of Jats and 11 closely related populations.

**Population**	**Rst distance**	**Haplotypes**	
FYR Macedonia	0.2128	509	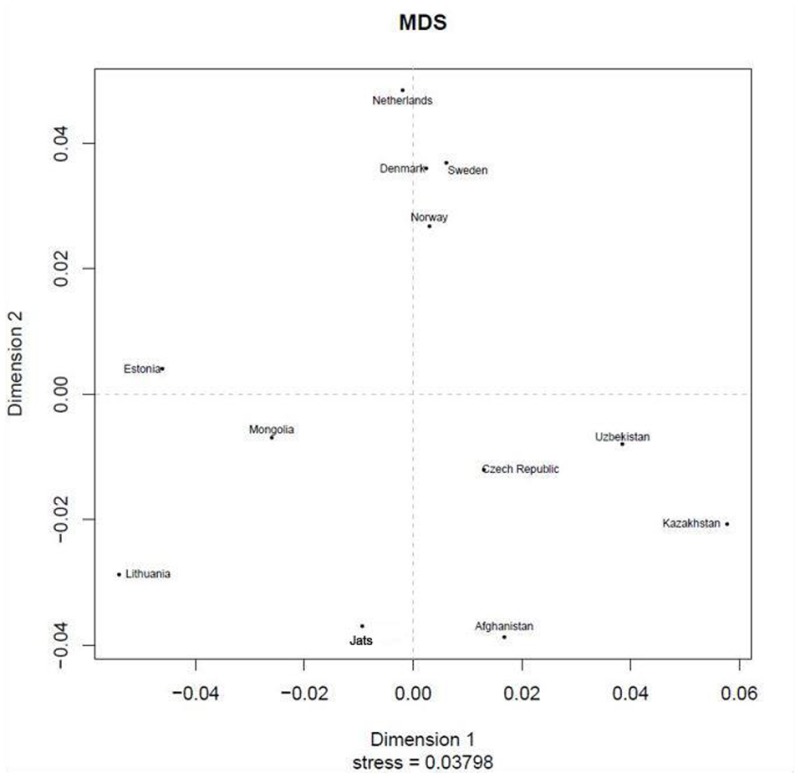
Croatia	0.2013	1,474	
Greece	0.1982	1,031	
China(Guangdong.Han)	0.1897	994	
Syria	0.1787	213	
Hungary	0.1478	1,448	
Finland	0.1449	1,126	
Iran	0.1394	973	
Turkey	0.1307	2,153	
Ukraine	0.1176	801	
Iraq	0.1097	249	
Azerbaijan	0.1018	117	
America	0.1012	100	
Sweden	0.0792	740	
Norway	0.0791	1,570	
China(Xinjiang, Kazakh)	0.0717	115	
Netherlands	0.0684	2,293	
Uzbekistan	0.0650	174	
Kazakhstan	0.0636	394	
Czec Republic	0.0595	2,324	
Lithuania	0.0558	380	
Estonia	0.0499	184	
Denmark	0.0477	275	
Mongolia	0.0471	780	
Afghanistan	0.0257	482	

The close genetic affinity between the Jats and the Afghani population was evident because most migrations and invasions into north India have passed through this territory. In the past, several Jat tribes and clans have inhabited parts of Afghanistan (Bellew, 1891).

### Haplogroups and geographic origins

The results of haplogroup analyses revealed that MRCAs of 302 Jats in our dataset belonged to nine different haplogroups—E, G, H, I, J, L, Q, R, and T—with nine different geographic origins.

The same nine haplogroups were used to compare the Jats with other ethnic groups of the Indian subcontinent. The haplogroups of 302 Jats and 1,855 other men in 38 ethnic groups of Bangladesh, India, and Pakistan are displayed in Table 2.

**Table 2:** Representation in nine Y-Chromosome haplogroups: Jats and thirty-eight ethnic groups of Indian subcontinent.

**Ethnic groups**	**n**	**E**	**G**	**H**	**I**	**J**	**L**	**Q**	**R**	**T**
Bangladesh, Bangladeshi	17			√		√			√	
Bangladesh, Bengali	43		√	√		√	√	√	√	
India, Agharia	10			√		√			√	
India, Ambalakarar	27		√	√		√	√		√	
India, Bhargavas	76			√		√			√	
India, Brahmin	126	√	√	√		√	√		√	
India, Chamar	17			√					√	
India, Chaturvedis	68			√		√	√		√	
India, Ezhava	113	√	√	√	√	√	√	√	√	√
India, Gujarati	107			√		√	√	√	√	
India, Irula	20			√		√	√		√	
India, Iyengar	42		√	√		√	√	√	√	
India, Iyer	48		√	√		√	√	√	√	
India, Jats	294	√	√	√	√	√	√	√	√	√
India, Kamar	16			√			√			
India, Kashmiri	21			√		√	√		√	
India, Koknasth Brahmin	25			√		√	√		√	
India, Konkane	50		√	√		√	√	√	√	√
India, Kurumba	17			√			√		√	
India, Lingayat	93		√	√		√	√		√	
India, Lodha	19			√		√			√	
India, Malani	30			√		√	√		√	
India, Malayali	95		√	√	√	√	√	√	√	√
India, Maratha	86		√	√		√	√	√	√	
India, Nair	47	√	√	√	√	√	√	√	√	√
India, Pallan	26			√		√	√		√	
India, Rajput	40			√		√	√		√	
India, Vanniyar	20			√		√	√		√	
India, Vellalar	32	√		√	√	√	√		√	
India, Vokkaliga	84		√	√		√	√		√	
Pakistan, Balochi	29	√		√		√	√		√	
Pakistan, Brahui	23		√	√		√	√		√	
Pakistan, Burusho	17		√	√		√	√		√	
Pakistan, Hazara	13				√	√		√	√	
Pakistan, Jats	8			√		√	√		√	
Pakistan, Kalash	20		√	√		√	√		√	
Pakistan, Makrani	20	√				√	√	√	√	
Pakistan, Pashtun	20			√		√	√	√	√	
Pakistan, Pathan	258		√	√		√	√	√	√	√
Pakistan, Sindhi	40	√	√	√		√	√	√	√	
Total Jats	302	3	11	11	3	29	111	47	86	1
Total others	1855	13	75	340	13	260	216	44	865	29
Total all groups	2157	16	86	351	16	289	327	91	951	30
Percent Jats	100.0%	1.0%	3.6%	3.6%	1.0%	9.6%	36.8%	15.6%	28.5%	0.3%
Percent others	100.0%	0.7%	4.0%	18.3%	0.7%	14.0%	11.6%	2.4%	46.6%	1.6%
Percent all groups	100.0%	0.7%	4.0%	16.3%	0.7%	13.4%	15.2%	4.2%	44.1%	1.4%

*Henke et al. (2001); Sengupta et al. (2006); Nagy et al. (2007); Zhao et al. (2009); Giroti and Talwar (2010); Nair et al. (2011); Chennakrishnaiah et al. (2013); Lee et al. (2014); Genographic Project (Genographic, 2016)*.

The results signified that the Jats shared an underlying genetic unity with several other ethnic communities in the Indian subcontinent with the same MRCAs and geographic origins. About 90% of the Jats and about 75% of the other 38 groups in the study belonged to the same four haplogroups J, L, Q, and R.

The geographic origins of the Jats in our study are summarized in Table 3.

**Table 3:** Ancestral geographic origins of 9 Y-chromosome haplogroups of the Jats.

**Haplogroup**	**India**	**Pakistan**	**Total**	**Percent**	**Marker**	**Age (kya)**	**Geographic origins**
E	3	0	3	1.0%	M96	~30–40	Northeast Africa, part of second migration out of Africa, initially settled in Middle East
G	11	0	11	3.6%	M201	~10–23	Eastern edge of the Middle East, close to Himalayan foothills, Indus Valley
H	10	1	11	3.6%	M69	~30	South central Asia, known as the “Indian Marker”
I	3	0	3	1.0%	M170	~25	Europe, Near East, Central Asia, known as the “European haplogroup”
J	28	1	29	9.6%	M304	~15	Fertile Crescent (Mesopotamia, the land in and around the Tigris and Euphrates rivers)
L	110	1	111	36.8%	M11	~25–30	Pamir Knot region (Hindu Kush, Tian Shan, Himalayas) in Tajikistan, Indus Valley
Q	47	0	47	15.6%	M242	~15–20	Siberia (North Asia), descendants first arrivals in North America
R	81	5	86	28.5%	M207	~4–27	Central Asia (Caspian Sea to border of western China), Kazakhstan, Uzbekistan, Turkmenistan
T	1	0	1	0.3%	M184	~25	Low frequencies Europe, the Middle East, North Africa, and East Africa
Totals	294	8	302	100.0%			

*(National Geographic Society's Genographic Project; Smolenyak and Turner, 2004; Wells, 2007; ISOGG, http://isogg.org/tree/index.html) kya = thousand years ago*.

**Figure 1:**
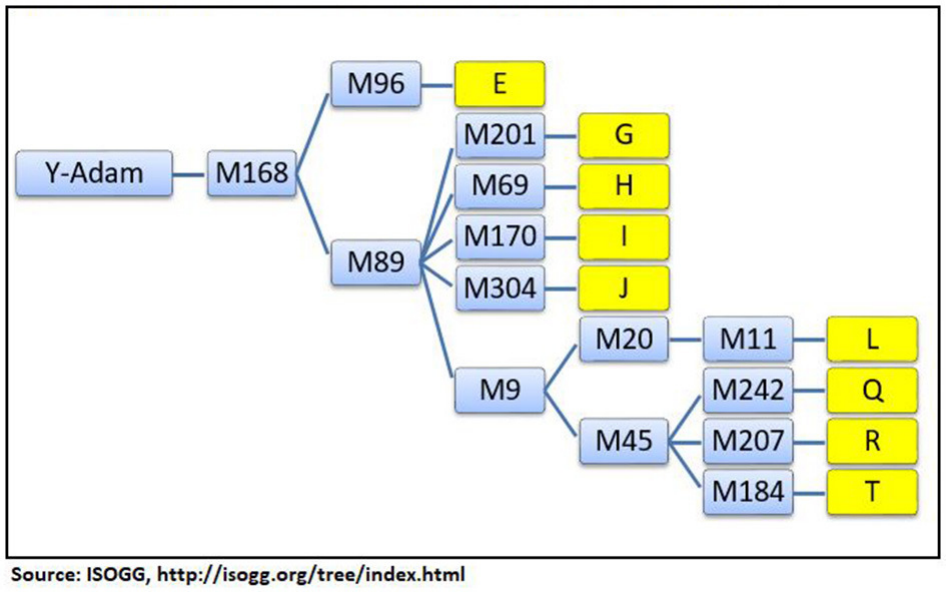
Phylogenetic tree of 9 Y-chromosome haplogroups of the Jats.

A short phylogenetic tree of nine haplogroups of the Jats in this study—with their key top-level markers starting from Y-Adam—appears in Figure 1.

### Haplogroup L (36.8%)

This is the largest haplogroup in the Jat sample population. It is present in the Indian population at an overall frequency of about 7–15% (Basu et al., 2003; Cordaux et al., 2004). Genetic studies suggest that this may be one of the original haplogroups of the creators of Indus Valley Civilization (McElreavey and Quintana-Murci, 2005; Sengupta et al., 2006). It has a frequency of about 28% in western Pakistan and Baluchistan, from where the agricultural creators of this civilization emerged (Qamar et al., 2002). The origins of this haplogroup can be traced to the rugged and mountainous Pamir Knot region in Tajikistan (Wells, 2007).

### Haplogroup R (28.5%)

This haplogroup originated in north Asia about 27,000 years ago (ISOGG, 2017). It is one of the most common haplogroups in Europe, with its branches reaching 80% of the population in some regions. One branch is believed to have originated in the Kurgan culture, known to be the first speakers of the Indo-European languages and responsible for the domestication of the horse (Smolenyak and Turner, 2004). From somewhere in central Asia, some descendants of the man carrying the M207 mutation on the Y chromosome headed south to arrive in India about 10,000 years ago (Wells, 2007). This is one of the largest haplogroups in India and Pakistan. Of its key subclades, R2 is observed especially in India and central Asia.

### Haplogroup Q (15.6%)

With its origins in central Asia, descendants of this group are linked to the Huns, Mongols, and Turkic people. In Europe it is found in southern Sweden, among Ashkenazi Jews, and in central and Eastern Europe such as, the Rhône-Alpes region of France, southern Sicily, southern Croatia, northern Serbia, parts of Poland and Ukraine. A subclade of this haplogroup is associated with Native American populations, and the mutation occurred 8 to 12 thousand years ago during the migration to the Americas through the Bering Strait (Smolenyak and Turner, 2004). It is estimated that as few as twenty people may have founded the initial native population of the Americas (Liu, 2016).

### Haplogroup J (9.6%)

The ancestor of this haplogroup was born in the Middle East area known as the Fertile Crescent, comprising Israel, the West Bank, Jordon, Lebanon, Syria, and Iraq. Middle Eastern traders brought this genetic marker to the Indian subcontinent (Kerchner, 2013).

### Haplogroups E, G, H, I, T (9.5%)

The ancestors of the remaining five haplogroups E, G, H, I, and T can be traced to different parts of Africa, Middle East, South Central Asia, and Europe (ISOGG, 2016).

## Discussion

### Sample size

In statistical analyses, as the population increases in size, the sample size increases at a diminishing rate, and remains relatively constant when it reaches a size of 380 or more. At about 384, the sample is generally representative for a population of one million, or more (Krejcie and Morgan, 1970). Ideally, the sample size should be 380, and preferably larger.

The dataset of 302 Jats used in our research represents a margin of error of 5.7% at a confidence level of 95%. In other words, if a survey is conducted one hundred times among a similar group of people (i.e., 302 × 100; 32,000 people in total), the distribution in haplogroups is expected to be about the same as in this study, with a margin of error of plus or minus 5.7%.

Although the sample of 302 records used in this research revealed key haplogroups for the Jats, the results are not representative of this entire ethnic group of an estimated 123 million people. It is already noted that the Muslim Jats of Pakistan were underrepresented in this study. A larger sample of Muslim Jats is likely to reveal a few additional haplogroups and provide a more complete picture. Therefore, to ascertain a representative distribution of haplogroups for the entire ethnic group of the Jats, the sample size should be at least 380, with a proportional representation of Hindus, Sikhs, and Muslims.

### Potential errors in haplogroup prediction

Because of the need for precision in matters relating to criminal and civil laws, the forensic genetics community is generally not in favor of determining haplogroups with STR profiles. It is held that STR haplotypes are not always identical by descent, but also identical by state, and can be rooted in different haplogroups.

A study that used STR profiles of 119 males in Argentina to determine haplogroups with two software programs—Whit Athey's Haplogroup Predictor (used in this study), and a Haplogroup Classifier developed at the University of Arizona—showed that the results were not totally accurate (Muzzio et al., 2011). Another study of 165 males in Nicaragua showed that Athey's Haplogroup Predictor produced accurate results for 95.2% of the sample, but 4.8% of the results were inaccurate (Nunez et al., 2012). For greater reliability in identifying Y chromosomal haplogroups, the forensic community's preferred method is to analyze (SNPs) on the Y chromosome in the lab with actual DNA samples.

Athey has explained that the main drawback of the haplogroup prediction method in his software is the size of the database of some Y-STR haplotypes from which the allele frequencies are calculated. For most haplogroups there is sufficient Y-STR haplotype data. However, for some haplogroups, such as, C, H, L, N, and Q, the database of Y-STR haplotypes is smaller, and the results may be prone to error (Athey, 2006).

Of the 302 records used in this study, 258 were processed through Whit Athey's software. Of these, 169 haplotypes belonged to the potentially error-prone haplogroups H, L, and Q, identified by Athey. Assuming an error rate of 5% for this software, as reported in the Nicaraguan study (Nunez et al., 2012), only 13 haplotypes (5% of 258) may have identified incorrect haplogroups, representing a potential error rate of about 4.3% (13/302) in the total sample used in this study. This suggests an accuracy of about 96% in the haplogroups and geographic origins identified in this study.

### Population mixture leading to endogamy

Studies have shown that most ethnic groups of the Indian subcontinent descended from a mixture of two divergent populations. These were the Ancestral North Indians (ANI) who were related to Central Asians, Middle Easterners, Caucasians, and Europeans, and the Ancestral South Indians (ASI) who were not closely related to any groups outside the subcontinent (Reich et al., 2009). These findings explain that admixture was widespread at one time (Moorjani et al., 2013).

The results of the AMOVA and MDS tests in our study confirmed that the Jats had genetic contributions from several populations in the Middle East, Central Asia, and Europe. After the arrival of people called Indo-Aryans—also known as Indo-European speakers—in north India about 2000 BCE, the caste system was introduced, and a stratified social hierarchy evolved. The upper-caste populations started practicing and encouraging endogamy about 70 generations (more than 2,000 years) ago (Basu et al., 2016). Another study suggested that endogamy started much later, about the time of foreign invasions in north India (Vadivelu, 2016).

Consanguinity is another form of endogamy. The word *consanguinity* comes from the Latin *con*, meaning shared, and *sanguis*, meaning blood. Marriage between people who have at least one recent common ancestor is known as consanguineous, and the children are considered inbred. Couples related as second cousins or closer account for an estimated 10.4% of the global population, with the highest rates in West, Central, and South Asia (Bittles and Black, 2010). According to the International Institute for Population Sciences in Mumbai, about 16% of marriages in India are consanguineous (Kuntla et al., 2013). In Pakistan, where first cousin marriages have occurred for generations, the rate is 67% (Yaqoob et al., 1993).

The motivation behind consanguinity is usually to keep bonds, wealth, and property within a family. For this reason, there is a long list of cousin marriages among famous people (e.g., Albert Einstein, Charles Darwin, and others), and in royal families all over the world. Although endogamy has become the general norm in India, and consanguinity is practiced in some parts of the subcontinent, most ethnic groups—including the Jats—carry a blend of genetic components from different populations in the past.

### Languages and genetic diversity

There are several thousand ethnic and tribal groups in the Indian subcontinent (Papiha, 1996; Xing et al., 2010). Members of these communities share common self-identities that are based on languages, customs, cuisines, and at least six major religions. There are 22 official languages and many dialects in the country (Annamalai, 2006). At least eight different languages—Balochi, Haryanvi, Hindi, Punjabi, Rajasthani, Saraiki, Sindhi, and Urdu—are spoken in the Jat communities, which demonstrates their genetic diversity.

### The Aryan-Scythian conundrum

The estimated population of the Indian subcontinent in 10,000 BCE was about 100,000 people, and stayed at this level until about 5,000 BCE, by when agriculture had spread in the Indus Valley (McEvedy and Jones, 1978). Since then the population has grown exponentially, with about 1.7 billion people in the Indian subcontinent now. Among the several thousand ethnic and tribal groups in the subcontinent, there are no existing population groups known as Indo-Aryan or Indo-Scythian. These appear to be labels that have been loosely applied to people who arrived in north India in a series of waves over a long period in the distant past.

Sir Risley's ethnographic classifications of Indian people did not provide any clues about the origins of the Indo-Aryans and the Scytho-Dravidians. But his studies showed that these two groups were physically different. According to the Imperial Gazetteer of India, the Indo-Scythians were likely pushed toward the south by the Indo-Aryans, mingled with the Dravidian population, and became the ancestors of an entirely different ethnic group known as the Marathas (Gazetteer, 1931).

The Pamir Knot region—from where the MRCA of haplogroup L emerged—is also the home of the Bactria-Margiana Archaeological Complex (BMAC), in a site called Gonur that represents a Bronze Age culture known as the Oxus civilization (Sarianidi, 2007). This BMAC site of around 4000 BCE was discovered and named by the Soviet archeologist Viktor Sarianidi. Among his findings, Sarianidi discovered evidence of sacred alters; traces of ingredients such as, poppy seeds, cannabis, and ephedra, used for a drink called soma; horse sacrifices; four wheeled chariots; and other connections with the Aryans (Sarianidi, 2007; Wood, 2007). Some BMAC materials of this type have been found in the Indus Valley sites. Archaeologist J. P. Mallory from Queens University (Ireland), and Indologist Asko Parpola from the University of Helsinki (Finland), have suggested a connection between the Aryans and BMAC (Mallory, 1989; Parpola, 1999). Because the MRCA of haplogroup L emerged from the same geographical area as the people called the Aryans, there may be a genetic link between the two.

The haplotypes of 26 ancient human specimens from the Krasnoyarsk area in Siberia, dated from between the middle of the second millennium BCE to the fourth century CE (Scythian and Sarmatian timeframe), revealed that nearly all specimens belonged to R1a, a subclade of haplogroup R, which is thought to mark the eastward migration of early Indo-Europeans (Keyser et al., 2009). Another survey of 217 samples from Europe and Asia revealed that R1a1, another subclade of haplogroup R, was spread across Eurasia (Pamjav et al., 2012). Because the origins of haplogroup R can be traced to the same geographical area, there may be a genetic link with the ancient people called Scythians.

Studies have shown that the Hindu Kush area from where these groups migrated to the Indian subcontinent served as a confluence of gene flows from adjoining areas rather than a source of distinctly autochthonous populations (Cristofaro et al., 2013). These people also arrived in north India at different times. As noted earlier, members of haplogroup R arrived about 10,000 years ago, the Indo-Aryan migrations started about 2000 BCE, and the Indo-Scythians arrived much later, around 200 BCE. Because of their physical differences and the large gaps between their arrival times, it can be inferred that these groups were genetically different and not the same people.

This study has shown that the genetic origins of the Jats can be traced to at least nine and possibly more MRCA's, with nine different geographical origins that are spread thousands of miles apart (e.g., from the Fertile Crescent to Serbia). These nine MRCAs were genetically different. Therefore, any assertion that Jats are descendants of a single ancient population such as, the Indo-Aryans or Indo-Scythians cannot be supported. However, certain members of the Jat ethnic group who belong to haplogroups L and R—along with members of several other ethnic groups in the Indian subcontinent who belong to the same two haplogroups—are the most probable candidates to be linked to these ancient populations.

## Conclusion

The human Y-chromosome provides a powerful molecular tool for analyzing Y-STR haplotypes and determining their haplogroups which lead to the ancient geographic origins of individuals. For this study, the Jats and 38 other ethnic groups in the Indian subcontinent were analyzed, and their haplogroups were compared. Using genetic markers and available descriptions of haplogroups from the Y-DNA phylogenetic tree, the geographic origins and migratory paths of their ancestors were traced.

The study demonstrated that based on their genetic makeup, the Jats belonged to at least nine specific haplogroups, with nine different lines of ancestry and geographic origins. About 90% of the Jats in our sample belonged to only four different lines of ancestry and geographic origins. Therefore, attributing the origins of this entire ethnic group to loosely defined ancient populations such as, Indo-Aryans or Indo-Scythians represents very broad generalities and cannot be supported. The study also revealed that even with their different languages, religions, nationalities, customs, cuisines, and physical differences, the Jats shared their haplogroups with several other ethnic groups of the Indian subcontinent, and had the same common ancestors and geographic origins in the distant past. Based on recent developments in DNA science, this study provided new insights into the ancient geographic origins of this major ethnic group in the Indian subcontinent. A larger dataset, particularly with more representation of Muslim Jats, is likely to reveal some additional haplogroups and geographical origins for this ethnic group.

## Ethics statement

This study presented in the manuscript does not involve human or animal subjects. All data used in the study are from existing databases and published sources, which are cited.

## Author contributions

DM analyzed data and wrote the paper; IM wrote the paper.

### Conflict of interest statement

The authors declare that the research was conducted in the absence of any commercial or financial relationships that could be construed as a potential conflict of interest.
