# Supplementary material for: Y-STR Haplogroup Diversity in the Jat Population Reveals Several Different Ancient Origins
Source: Front Genet. 2017 Sep 20;8:121. doi: 10.3389/fgene.2017.00121 (PMC5611447; doi:10.3389/fgene.2017.00121)
Supplement: Supplementary file 1 [file DataSheet1.PDF]

Country data sources used at YHRD.org for AMOVA and MDS analyses

Afghanistan: Algenas C and Tillmar AO. 2014. Population genetics of 29 autosomal STRs and 17 Y-chromosomal sample from Afghanistan. *International Journal of Legal Medicine*. 128(2), 279-280; Lacau H, Bukhari A, Gayden T, Salvia L, Regueiro M, Stojkovic O, et al. 2011. Y-STR profiling in two Afghanistan populations. *Legal Medicine (Tokyo)*. 13(2), 103-8; Achakzai NM, Rahman Z, Shahzad MS, Daud S, Zar MS, Israr M, et al. 2012. Y-chromosomal STR analysis in the Pashtun population of Southern Afghanistan. *Forensic Science International Genetics*. 6(4), e103-5

Armenia: Nasidze I, Schadlich H, Stoneking M. 2003. Haplotypes from the Caucasus, Turkey and Iran for nine Y-STR loci. *Forensic Science International*. 137(1), 85-93

Azerbaijan: Nasidze I, Schadlich H, Stoneking M. 2003. Haplotypes from the Caucasus, Turkey and Iran for nine Y-STR loci. *Forensic Science International*. 137(1), 85-93; Roewer L, Willuweit S, Stoneking M, Nasidze I. 2009. A Y-STR database of Iranian and Azerbaijanian minority populations. *Forensic Science International Genetics*. 4(1), e53-5

China, Guandong, Han: Wang Y, Zhang Y, Zhang C, Li R, Yang Y, Ou X, et al. 2016. Genetic polymorphisms and mutation rates of 27 Y-chromosomal STRs in a Han population from Guangdong Province, Southern China. *Forensic Science International: Genetics*. 21, 5-9

China, Xinjiang, Kazakh: Shan W, Ablimit A, Zhou W, Zhang F, Ma Z, Zheng X. 2014. Genetic polymorphism of 17 Y chromosomal STRs in Kazakh and Uighur populations from Xinjiang, China. *International Journal Legal Medicine*. 128(5), 743-4

Croatia: Roewer L, Croucher PJ, Willuweit S, Lu TT, Kayser M, Lessig R, et al. 2005. Signature of recent historical events in the European Y-chromosomal STR haplotype distribution. *Human Genetics*. 116(4), 279-91; Grskovic B, Mrsic G, Polasek O, Vrdoljak A, Merkas S, Andelinovic S. 2011. Population data for 17 short tandem repeat loci on Y chromosome in northern Croatia. *Molecular Biology Reports*. 38(3), 2203-9

Czech Republic: Zastera J, Roewer L, Willuweit S, Skerka P, Benesova L, Minarik M. 2010. Assembly of a large Y-STR haplotype database for the Czech population and investigation of its substructure. *Forensic Science International Genetics*. 4(3), e75-8

Denmark: National/Population Database, YHRD.org

Estonia and Lithuania: Lessig R, Edelmann J. 2001. Population data of Y-chromosomal STRs in Lithuanian, Latvian and Estonian males. *Forensic Science International*. 120(3), 223-5

Finland: Hedman M, Pimenoff V, Lukka M, Sistonen P, Sajantila A. 2004. Analysis of 16 Y STR loci in the Finnish population reveals a local reduction in the diversity of male lineages. *Forensic Science International*. 142(1), 37-43

Greece: Parreira KS, Lareu MV, Sanchez Diz P, Skitsa I, Carracedo A. 2002. DNA typing of short tandem repeat loci on Y-chromosome of Greek population. *Forensic Science International*. 126(3), 261-4; Kovatsi L, Saunier JL, Irwin JA. 2009. Population genetics of Y-chromosome STRs in a population of Northern Greeks. *Forensic Science International Genetics*. 4(1), e21-2; Katsaloulis P, Tsekoura K, Vouropoulou M, Miniati P. 2013. Genetic population study of 11 Y chromosome STR loci in Greece. *Forensic Science International Genetics*. 7(3), e56-8; Bosch E, Calafell F, Gonzalez Neira A, Flaiz C, Mateu E, Schell HG, et al. 2006. Paternal and maternal lineages in the Balkans show a homogeneous landscape over linguistic barriers, except for the isolated Aromuns. *Annals Human Genetics.* 70(Pt 4), 459-87; Robino C, Varacalli S, Gino S, Chatzikyriakidou A, Kouvatsi A, Triantaphyllidis C, et al. 2004. Y-chromosomal STR haplotypes in a population sample from continental Greece, and the islands of Crete and Chios. *Forensic Science International*. 145(1), 61-4

Hungary: Volgyi A, Zalan A, Szvetnik, E, Pamjav H. 2009. Hungarian population data for 11 Y-STR and 49 Y-SNP markers. *Forensic Science International Genetics*. 3(2), e27-8; Nagy, Melinda, Lotte Henke, Jürgen Henke, Prasanta K. Chatthopadhyay, Antónia Völgyi, and Andrea Zalán, et al. 2007. Searching for the origin of Romanies: Slovakian Romani, Jats of Haryana and Jat Sikhs Y-STR data in comparison with different Romani populations. *Forensic Science International* 169 (1):19-26. doi: 10.1016/j.forsciint.2006.07.020.; Furedi S, Woller J, Padar Z,

Angyal M. 1999. Y-STR haplotyping in two Hungarian populations. *International Journal of Legal Medicine*. 113(1), 38-42; Beer Z, Csete K, Varga T. 2004. Y-chromosome STR haplotype in Szekely population. *Forensic Science International*. 139(2-3), 155-8; Egyed B, Furedi S, Angyal M, Boutrand L, Vandenberghe A, Woller J, et al. 2000. Analysis of eight STR loci in two Hungarian populations. *International Journal of Legal Medicine*. 113(5), 272-5

Iran: Alshamali F, Pereira L, Budowle B, Poloni ES, Currat M. 2009. Local population structure in Arabian Peninsula revealed by Y-STR diversity. *Human Heredity*. 68(1), 45-54; Nasidze I, Schadlich H, Stoneking M. 2003. Haplotypes from the Caucasus, Turkey and Iran for nine Y-STR loci. *Forensic Science International*. 137(1), 85-93; Roewer L, Willuweit S, Stoneking M, Nasidze I. 2009. A Y-STR database of Iranian and Azerbaijanian minority populations. *Forensic Science International Genetics*. 4(1), e53-5

Iraq: National/Population Database, YHRD.org

Kazakhstan: National/Population Database**,** YHRD.org

Macedonia: Jakovski Z, Nikolova K, Jankova Ajanovska R, Marjanovic D, Pojskic N, Janeska B. 2011. Genetic data for 17 Y-chromosomal STR loci in Macedonians in the Republic of Macedonia. *Forensic Science International Genetics*. 5(4), e108-11; Spiroski M, Arsov T, Kruger C, Willuweit S, Roewer L. 2005. Y-chromosomal STR haplotypes in Macedonian population samples. *Forensic Science International*. 148(1), 69-73; Bosch E, Calafell F, Gonzalez Neira A, Flaiz C, Mateu E, Schell HG, et al. 2006. Paternal and maternal lineages in the Balkans show a homogeneous landscape over linguistic barriers, except for the isolated Aromuns. *Annals Human Genetics.* 70(Pt 4), 459-87

Mongolia: Kayser M, Caglia A, Corach D, Fretwell N, Gegrig C, Graziosi G, et al., 1997. Evaluation of Y-chromosomal STRs: a multicenter study. *International Journal Legal Medicine*. 110(3), 125-33; Keyser C, Crubezy E, Pamzsav H, Varga T, Ludes B. 2006. Population origins in Mongolia: genetic structure analysis of ancient and modern DNA. *American Journal of Physical Anthropology*. 131(2), 272-81; Kim YJ, Shin DJ, Kim JM, Jin HJ., Kwak KD, Han MS,

et al. 2001. Y-chromosome STR haplotype profiling in the Korean population. *Forensic Science International*. 115(3), 231-7

Netherlands: National/Population Database**,** YHRD.org

Norway: Dupoy BM, Andreassen R, Flones AG, Tomassen K, Egeland T, Brion M, et al. 2001. Y-chromosome variation in a Norwegian population sample. *Forensic Science International*. 117(3), 163-73; Dupoy BM, Stenersen M, Lu TT, Olaisen B. 2006. Geographic heterogeneity of Y-chromosomal lineages in Norway. *Forensic Science International*. 164(1)

Sweden: Holmlund G, Nilsson H, Karlsson A, Lindblom B. 2006. Y-chromosome STR haplotypes in Sweden. *Forensic Science International*. 160(1), 66-79; Karlsson AO, Wallerstrom T, Gotherstrom A, Holmlund G. 2006. Y-chromosome diversity in Sweden – a long time perspective. *European Journal of Human Genetics*. 14(8), 963-70

Syria: Donbak L, Bajanowski T, Brinkmann B, Hohoff C. 2006. Y-STR haplotypes in populations from the Eastern Mediterranean region of Turkey. *International Journal Legal Medicine*. 120(6), 395-6

Turkey: Serin A, Canan H, Alper B, Sertdemir Y. 2011. Haplotype frequencies of 17 Y-chromosomal short tandem repeat loci from the Cukurova region of Turkey. *Croatian Medical Journal*. 52(6), 703-B; Henke J, Henke L, Chatthopadhyay P, Kayser M, Dulmer M, Cleef S, Poche H, Felske Zech H. 2001. Application of Y-chromosomal STR haplotypes to forensic genetics. *Croatian Medical Journal*. 42(3), 292-7; Cakir AH, Celebioglu A, Yardimci E. 2004. Y-STR haplotypes in Central Anatolia region of Turkey. *Forensic Science International*. 144(1), 59-64; Donbak L, Bajanowski T, Brinkmann B, Hohoff C. 2006. Y-STR haplotypes in populations from the Eastern Mediterranean region of Turkey. *International Journal Legal Medicine*. 120(6), 395-6; Brinkmann C, Forster P, Schurenkamp M, Horst J, Rolf B, Brinkmann B. 1999. Human Y-chromosomal STR haplotypes in a Kurdish population sample. *International Journal Legal Medicine.* 112(3), 181-3; Ozbas Gerceker F, Bozman N, Arsian A, Serin A. 2013. Population Data for 17 Y-STRs in Samples from Southeastern Anatolia Region of Turkey. *International Journal Human Genetics*. 13(2), 105-111; Nasidze I, Schadlich H, Stoneking M.

2003. Haplotypes from the Caucasus, Turkey and Iran for nine Y-STR loci. *Forensic Science International*. 137(1), 85-93; Alakoc YD, Gokcumen O, Tug A, Gultekin T, Gulec E, Schurr TG. 2010. Y-chromosome and autosomal STR diversity in four proximate settlements in Central Anatolia. *Forensic Science International Genetics*. 4(5), e135-7

Ukraine: Mielnik Sikorska M, Daca P, Wozniak M, Malyarchuk BA, Bednarek J, Dobosz T, et al. 2013. Genetic data from Y chromosome STR and SNP loci in Ukraine population. *Forensic Science International Genetics.* 7(1), 200-3

Uzbekistan:

National/Population Database**,** YHRD.org
